# Supplementary material for: Modeling glioblastoma heterogeneity as a dynamic network of cell states
Source: Mol Syst Biol. 2021 Sep 16;17(9):e10105. doi: 10.15252/msb.202010105 (PMC8444284; doi:10.15252/msb.202010105)
Supplement: Supplementary file 5 — Source Data for Figure 3 [file MSB-17-e10105-s001.zip › Figure3A_sourcedata/GSEA_3065/hallmarks_state4.GseaPreranked.1623416296068/gsea_report_for_na_neg_1623416296068.html]

Report for na\_neg 1623416296068 [GSEA]

| GS  follow link to MSigDB | GS DETAILS | SIZE | ES | NES | NOM p-val | FDR q-val | FWER p-val | RANK AT MAX | LEADING EDGE || 1 | HALLMARK\_E2F\_TARGETS | Details ... | 164 | -0.81 | -3.10 | 0.000 | 0.000 | 0.000 | 783 | tags=74%, list=13%, signal=82% |
| 2 | HALLMARK\_G2M\_CHECKPOINT | Details ... | 150 | -0.81 | -3.04 | 0.000 | 0.000 | 0.000 | 813 | tags=68%, list=13%, signal=76% |
| 3 | HALLMARK\_MITOTIC\_SPINDLE | Details ... | 138 | -0.61 | -2.30 | 0.000 | 0.000 | 0.000 | 801 | tags=36%, list=13%, signal=40% |
| 4 | HALLMARK\_MYC\_TARGETS\_V1 | Details ... | 173 | -0.56 | -2.16 | 0.000 | 0.000 | 0.000 | 1654 | tags=57%, list=26%, signal=75% |
| 5 | HALLMARK\_MTORC1\_SIGNALING | Details ... | 137 | -0.56 | -2.09 | 0.000 | 0.000 | 0.000 | 1248 | tags=47%, list=20%, signal=57% |
| 6 | HALLMARK\_MYC\_TARGETS\_V2 | Details ... | 30 | -0.68 | -2.01 | 0.000 | 0.000 | 0.000 | 1681 | tags=70%, list=27%, signal=95% |
| 7 | HALLMARK\_SPERMATOGENESIS | Details ... | 34 | -0.66 | -1.99 | 0.000 | 0.000 | 0.000 | 851 | tags=53%, list=14%, signal=61% |
| 8 | HALLMARK\_UNFOLDED\_PROTEIN\_RESPONSE | Details ... | 71 | -0.52 | -1.76 | 0.000 | 0.005 | 0.038 | 1009 | tags=35%, list=16%, signal=41% |
| 9 | HALLMARK\_EPITHELIAL\_MESENCHYMAL\_TRANSITION | Details ... | 100 | -0.49 | -1.76 | 0.000 | 0.005 | 0.040 | 1186 | tags=39%, list=19%, signal=47% |
| 10 | HALLMARK\_ANGIOGENESIS | Details ... | 19 | -0.65 | -1.73 | 0.008 | 0.007 | 0.062 | 169 | tags=21%, list=3%, signal=22% |
| 11 | HALLMARK\_ANDROGEN\_RESPONSE | Details ... | 60 | -0.48 | -1.62 | 0.009 | 0.024 | 0.229 | 1137 | tags=40%, list=18%, signal=48% |
| 12 | HALLMARK\_UV\_RESPONSE\_UP | Details ... | 79 | -0.45 | -1.54 | 0.007 | 0.043 | 0.403 | 1327 | tags=38%, list=21%, signal=48% |
| 13 | HALLMARK\_CHOLESTEROL\_HOMEOSTASIS | Details ... | 44 | -0.48 | -1.52 | 0.037 | 0.050 | 0.485 | 437 | tags=25%, list=7%, signal=27% |
| 14 | HALLMARK\_ESTROGEN\_RESPONSE\_LATE | Details ... | 84 | -0.43 | -1.51 | 0.011 | 0.049 | 0.498 | 1289 | tags=39%, list=21%, signal=49% |
| 15 | HALLMARK\_APOPTOSIS | Details ... | 82 | -0.41 | -1.45 | 0.025 | 0.075 | 0.663 | 491 | tags=20%, list=8%, signal=21% |
| 16 | HALLMARK\_INFLAMMATORY\_RESPONSE | Details ... | 59 | -0.40 | -1.36 | 0.053 | 0.149 | 0.900 | 1312 | tags=32%, list=21%, signal=40% |
| 17 | HALLMARK\_GLYCOLYSIS | Details ... | 111 | -0.37 | -1.35 | 0.048 | 0.147 | 0.906 | 1515 | tags=35%, list=24%, signal=46% |
| 18 | HALLMARK\_APICAL\_SURFACE | Details ... | 17 | -0.52 | -1.33 | 0.133 | 0.163 | 0.939 | 1095 | tags=41%, list=17%, signal=50% |
| 19 | HALLMARK\_DNA\_REPAIR | Details ... | 99 | -0.35 | -1.26 | 0.102 | 0.249 | 0.989 | 978 | tags=26%, list=16%, signal=31% |
| 20 | HALLMARK\_IL6\_JAK\_STAT3\_SIGNALING | Details ... | 25 | -0.45 | -1.25 | 0.209 | 0.252 | 0.992 | 89 | tags=8%, list=1%, signal=8% |
| 21 | HALLMARK\_PI3K\_AKT\_MTOR\_SIGNALING |  | 65 | -0.36 | -1.19 | 0.194 | 0.332 | 0.999 | 270 | tags=9%, list=4%, signal=10% |
| 22 | HALLMARK\_KRAS\_SIGNALING\_UP |  | 66 | -0.34 | -1.14 | 0.260 | 0.422 | 1.000 | 277 | tags=12%, list=4%, signal=13% |
| 23 | HALLMARK\_ESTROGEN\_RESPONSE\_EARLY |  | 76 | -0.31 | -1.07 | 0.359 | 0.565 | 1.000 | 1289 | tags=30%, list=21%, signal=38% |
| 24 | HALLMARK\_ALLOGRAFT\_REJECTION |  | 46 | -0.34 | -1.06 | 0.377 | 0.552 | 1.000 | 1179 | tags=28%, list=19%, signal=35% |
| 25 | HALLMARK\_COAGULATION |  | 50 | -0.32 | -1.02 | 0.427 | 0.626 | 1.000 | 186 | tags=10%, list=3%, signal=10% |
| 26 | HALLMARK\_HEDGEHOG\_SIGNALING |  | 22 | -0.37 | -1.01 | 0.431 | 0.627 | 1.000 | 679 | tags=18%, list=11%, signal=20% |
| 27 | HALLMARK\_COMPLEMENT |  | 76 | -0.29 | -1.01 | 0.463 | 0.609 | 1.000 | 453 | tags=11%, list=7%, signal=11% |
| 28 | HALLMARK\_HYPOXIA |  | 100 | -0.26 | -0.95 | 0.552 | 0.712 | 1.000 | 949 | tags=20%, list=15%, signal=23% |
| 29 | HALLMARK\_IL2\_STAT5\_SIGNALING |  | 84 | -0.27 | -0.95 | 0.547 | 0.694 | 1.000 | 1867 | tags=45%, list=30%, signal=64% |
| 30 | HALLMARK\_WNT\_BETA\_CATENIN\_SIGNALING |  | 16 | -0.38 | -0.94 | 0.550 | 0.676 | 1.000 | 57 | tags=6%, list=1%, signal=6% |
| 31 | HALLMARK\_FATTY\_ACID\_METABOLISM |  | 78 | -0.25 | -0.88 | 0.664 | 0.783 | 1.000 | 493 | tags=12%, list=8%, signal=12% |
| 32 | HALLMARK\_XENOBIOTIC\_METABOLISM |  | 75 | -0.25 | -0.87 | 0.709 | 0.774 | 1.000 | 1039 | tags=20%, list=17%, signal=24% |
| 33 | HALLMARK\_APICAL\_JUNCTION |  | 82 | -0.24 | -0.86 | 0.742 | 0.782 | 1.000 | 1298 | tags=26%, list=21%, signal=32% |
| 34 | HALLMARK\_PROTEIN\_SECRETION |  | 78 | -0.24 | -0.82 | 0.790 | 0.817 | 1.000 | 1205 | tags=18%, list=19%, signal=22% |
| 35 | HALLMARK\_PEROXISOME |  | 55 | -0.24 | -0.79 | 0.812 | 0.854 | 1.000 | 630 | tags=11%, list=10%, signal=12% |
| 36 | HALLMARK\_NOTCH\_SIGNALING |  | 16 | -0.29 | -0.76 | 0.785 | 0.869 | 1.000 | 62 | tags=6%, list=1%, signal=6% |
Table: Gene sets enriched in phenotype **na **[plain text format]****
